# Liquid resistivity of pharmaceutical propellants using novel resistivity cell

**DOI:** 10.1038/s41598-023-45253-6

**Published:** 2023-11-05

**Authors:** Hussein Ahmad, Manoochehr Rasekh, Nadarajah Manivannan, Wamadeva Balachandran

**Affiliations:** College of Engineering, Design and Physical Sciences, Brunel University London, Uxbridge, UB8 3PH UK

**Keywords:** Biotechnology, Engineering

## Abstract

Metered-dose inhalers employ propellants to produce pharmaceutical aerosols for treating respiratory conditions like asthma. In the liquid phase, the DC volume resistivity of pharmaceutical propellants, including R134a, R152a, and R227ea, was studied at saturation pressures and room temperature (not vapour phase). These measurements are essential for industries like refrigerants. Aerosols from metered dose inhalers (MDIs) with these propellants become electrically charged, affecting medicament deposition in lung. The resistivity was measured using a novel concentric cylinder-type capacitance cell designed in-house. The resistivity for the propellants (R134a, R152a, and R227ea) was found to be 3.02 × 10^10^ Ωm, 2.37 × 10^9^ Ωm and 1.31 × 10^10^ Ωm, respectively. The electrical resistivity data obtained was found to be at least two orders of magnitude higher than the limited data available in the literature. Challenges in the resistivity cell’s development and performance are discussed, with a focus on various propellants and their mixtures with ethanol and moisture concentrations. The resistivity of propellant mixtures containing moisture concentrations ranging from 5 to 500 ppm and ethanol concentrations ranging between 1000 and 125,000 ppm was determined. The resistivity was tested across 10-min and 1-h periods and was performed in accordance with the contemporary IEC 60247 standard.

## Introduction

R134a (Tetrafluoroethane-C_2_H_2_F_4_), R227ea (Heptafluropropane, CF_3_CFHCF_3_), and R152a (Difluoroethane, C_2_H_4_F_2_) are hydrofluorocarbons (HFCs) widely used as propellants in the pharmaceutical industry for metered dose inhalers (MDI) to generate inhalable drug aerosols to treat respiratory conditions. Such HFCs were developed to overcome environmental issues associated with the use of chlorofluorocarbons (CFCs) and hydrochlorofluorocarbons (HCFCs), as the chlorine in those molecules is responsible for the depletion of the ozone layer. Besides their application in MDIs, they are also used as refrigerants^1,2^. It has been recognised that when these inhalers deliver the aerosols, they become electrically charged. Consequently, the medicament deposition in the human lungs is also influenced by the level and polarity of the charge. The resistivity of the propellant, which typically makes up more than 99% of the inhaled dose, is an important aspect for understanding the extent of charging in aerosols delivered by such devices^3^. Higher resistivity limit charge dissipation in the propellant, causing charge to accumulate^4^. Suspensions used in MDIs contain micron-sized drug particles suspended in the propellant, while in solution MDIs, the drug is dissolved in the propellant using a cosolvent. The main co-solvent in MDI formulations is ethanol to increase drug or excipient solubility^5^. A thorough understanding of these mechanisms requires systematically studying the materials and processes involved. Such an understanding may enable one to control the charge-to-mass ratio of the aerosol to achieve site-specific lung deposition of the active pharmaceutical ingredients (API).

In addition, dielectric liquids play a vital role in suppressing arcing and corona discharge, while also serving as coolants and electrical insulators. The determination of resistivity allows for the evaluation and testing of the performance of these liquids in high-voltage applications, such as transformers, capacitors, cables, and high-voltage switchgear^6^. Similarly, the use of hermetic and semi-hermetic compressors in refrigeration has led to the need for verifying the dielectric properties of refrigerants employed in these systems and exploring alternative options that require high resistivity^7^. Consequently, there is a growing demand for conducting resistivity tests across various operating conditions. Regrettably, existing commercial resistivity cells are tailored solely to the requirements of the power industry, neglecting the diverse chemical compositions of liquids investigated outside this industry. This limitation prompts academics and researchers to develop their own cells specifically suited for studying the resistivity of alternative liquids, particularly those operating under non-atmospheric pressures.

Some properties and the molecular structure of the aforementioned propellants are listed in Table 1. Several studies have been published previously by scientists to measure the resistivities of the propellants, with each study developing novel devices to perform the measurements.

**Table 1:** Properties of the propellants used in this study.

Propellant	Molecular structure	Relative permittivity	Density (kg/m^3^) at 25 °C	Viscosity (Pa s)
R134a	CH_2_FCF_3_	1.15–1.22	1206.7	0.0005
R152a	CH_3_CHF_2_	1.004	899	0.00031
R227ea	CF_3_CHFCF_3_	1.25–1.29	1590.6	0.00076

Values concerning the DC resistivity of R134a and R152a were first published by Fellows et al.^8^ and later by Feja^9^. Fellows tested the resistivities using both AC and DC voltages and measured the DC resistivity for R134a and R152a to be 6.6 × 10^8^ Ωm and 2.2 × 10^7^ Ωm, respectively. Feja^9^ tested several electrical properties, including the relative permittivity, dielectric dissipation factor, and DC resistivity, across a temperature range between − 30 and 90 °C, and tested mixtures with different concentrations of polyester oil. They found the DC resistivity of R134a and R152a to be 10^8^ Ωm and 10^7^ Ωm, respectively. The resistivity of R134a was investigated by Meurer^7^, followed by Dschung^10^. The latter also measured data for the resistivity of R227ea. Dschung^10^ investigated the DC resistivity using a novel device, finding that R134a’s and R227ea’s resistivities were 6 × 10^6^ Ωm and 1.3 × 10^8^ Ωm, respectively.

R152a possesses a global warming potential (GWP) substantially lower than the R134a and R227ea HFCs^11,12^. Therefore, R152a is considered a potential candidate to replace the two HFCs (R134a and R227ea) in different applications, particularly under the strong impetus of the EU F-gas regulation and the 2016 Kigali Amendment of the Montreal Protocol to phase down the global use of high-GWP HFCs^13,14^. Understanding the resistivity of R152a in relation to R134a and R227ea is, therefore, critical to further adopting R152a and determining its potential as an inhaler propellant. Moreover, R152a is a flammable substance; therefore, there is a risk of the propellant catching fire during the actuation of an inhaler if substantial charges develop, making it imperative that the resistivity is accurately determined to prevent such a scenario from occurring. Determining the volume resistivity of the propellants with mixtures of moisture and ethanol would also significantly help further our understanding of the properties of inhaler formulation and potentially improve them due to ethanol and moisture being widely used as cosolvents. The aim of this study is to design a novel resistivity cell capable of measuring high resistivities while ensuring the experimental process minimises contamination of samples to determine propellant resistivities with more precision and accuracy than previous studies. It is also to collect data on the propellants and their mixtures with ethanol and moisture with respect to their resistivity in the liquid phase at room temperature.

## Materials and methods

### Theory of electrical bulk resistivity

Volume resistivity is a measure of a dielectric liquid’s ability to resist electric current flow. It is denoted by the symbol ρ and has an SI unit of Ωm. The value reflects the presence or absence of free electrons, ions, and other conductive contaminants. It helps in assessing a material's deviation from its ideal dielectric characteristics^15^. The volume resistivity expressed by Eq. (1):

1 $$\rho = \mathop \sum \limits_{i} \frac{1}{{k_{i} q_{i} }}$$

where the medium possesses charge carriers of species $i$ with mobility ${k}_{i}$ and a volume charge density of ${q}_{i}$. The resistivity is representative when the liquid is in thermodynamic equilibrium, and external conditions do not impact the charge carriers’ mobility and density^16^. When these conditions are met, the dielectric behaves like an RC parallel circuit. Ohm's law governs its behaviour if the applied voltage doesn’t disrupt this equilibrium.

The relationship between resistivity, resistance, voltage, current, and the geometry of the test setup is expressed by:

2 $$\rho = \frac{V}{I}\left( \frac{A}{L} \right)$$

where R, Resistance; L, Distance between electrodes; V, Voltage; $\rho$, Resistivity; A, Cross-sectional area of material under test; I, Current

The factor $\left( \frac{L}{A} \right)$ is the cell constant (with SI unit /m^−1^). This constant convert measured resistance to resistivity. The accurate value of this constant is found experimentally using calibration liquids with known resistivity. This is essential because the electrode's geometrical surface area often differs from the electrochemical area where electron transfer occurs in liquid dielectrics^17^.

### Charge carrier mobility

The charge carrier’s mobility can be determined using the time-of-flight (ToF) method^18^. This is the time interval for the charge carriers to cross the interelectrode gap filled with the propellant. When a DC potential difference, V, is applied between the electrodes separated by a distance, L, the charge carriers will move in a uniform electric field, E, which is given by Eq. (3):

3 $$E = \frac{V}{L}$$

The propellants in liquid form under pressure behave as dielectric liquids. The charge carriers will be randomly distributed in the bulk of the liquid. When an external electric field stresses the liquid, the charge carriers will start moving across the electrode gap and manifest as electric current. This current can be measured using an electrometer connected in series with a liquid-filled gap. A single current peak will be registered if the dielectric liquid contains charge carriers with the same polarity. The magnitude of the drift velocity of the charge carriers, v, within the electrode gap, can be obtained as follows:

4 $$v = \frac{L}{ToF}$$

The drift velocity is also proportional to the field:

5 $$v = \mu \cdot E$$

where µ is the charge carrier mobility. Thus, µ can now be expressed by Eq. (6):

6 $$\mu = \frac{{L^{2} }}{ToF \cdot V}$$

where V is the applied potential.

Suppose the applied electric field is not strong enough to generate new charge carriers through ionisation or dissociation processes in the liquid or by injecting electrons from the cathode into the bulk liquid. In that case, the conduction current is defined by the movement of charge carriers that already exist in the liquid, and the current will demonstrate Ohmic behaviour; the current, I, is proportional to V.

### Design of the resistivity cell

#### Resistivity cell

**Figure 1:**
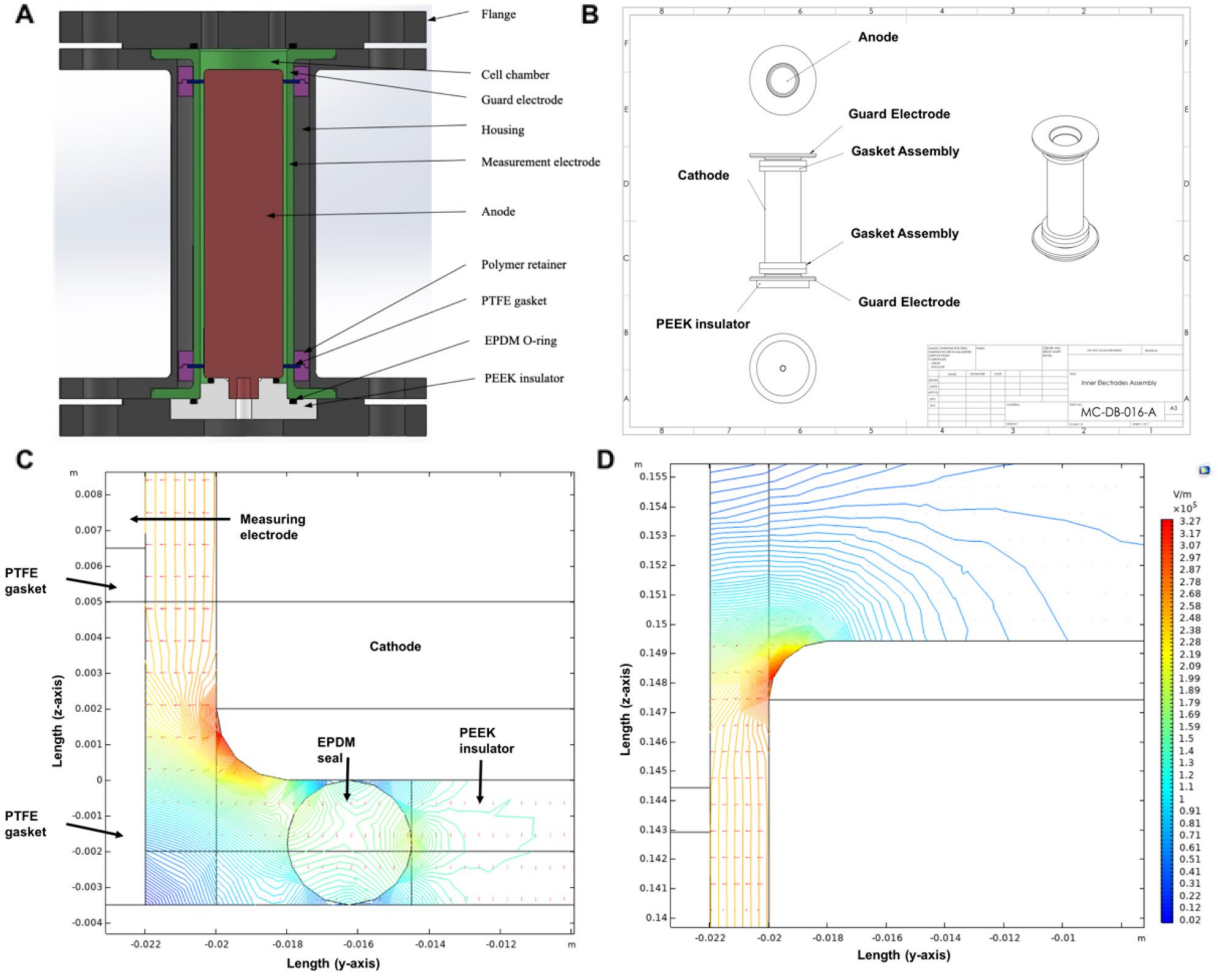
CAD drawings (2D) of resistivity cell components: (**A**) cross section of the complete resistivity cell and (**B**) internal components of the resistivity cell. COMSOL simulations of the electrical field strength across the bottom (**C**), and at the top (**D**) of the anode. The plots show 2D visualisations of the field strength in V/m with a 10^5^-scaling factor for clarity. The electric field is homogenous (parallel field lines) within the measuring area but distorts (curved lines) near the top and bottom edges.

The resistivity cell consists of concentric cylinders enclosed by an outer stainless-steel housing with custom stainless-steel flanges mounted to both ends (see Fig. 1A and B). EPDM (ethylene propylene diene monomer rubber) O-rings are installed between the flanges, outer housing, and cathode electrodes to form a hermetically sealed body. The electrodes are made of stainless steel. The measuring cathode electrode is suspended between guard cathode electrode cylinders at each axial end, with 1.5 mm-thick Sigma 500 polytetrafluoroethylene (PTFE) gaskets separating each of these cylinders. The outer housing diameter is 73 mm, the internal measuring electrode diameter is 44 mm, and the cathode diameter is 40 mm. There is a 2-mm separation between the anode and the cathode electrode, along with the attached gasket and guard cylinders. The cathode has a 2 mm radius of curvature at each edge to limit field enhancement resulting from sharp edges. The length of the measuring electrode was determined to be 136 mm. This was to ensure a low enough cell constant to ensure our cell possessed a suitable resistivity measurement range when testing the desired HFCs. The measuring electrode is in contact with three ground contacts that radially alternate by 120° and are fed through fittings at the side of the housing. Three contacts are used to prevent misalignment of the measuring electrode, with only one of the contacts used for measurement. The inlet port consists of a threaded hole connected to the bottle containing the propellant and connected to a vacuum pump and nitrogen source. An outlet port at the top flange is also established to evacuate the fluids after each test. The anode is connected to the voltage source through a machine screw at the bottom of the cylinder and rests on a polyether ether ketone (PEEK) insulator. The guard or cathode electrodes at each side of the measuring electrode limit fringe fields so that current virtually only flows radially to improve measurement accuracy^19^. The outer housing exists primarily to ensure the firm positioning of the cylindrical electrodes. It also serves to improve safety by protecting the user from the electrodes and shielding the signal from stray electric fields external to the cell that may affect the measurement.

The electrodes are constructed from stainless steel 316 (SS316) and subjected to electro-polishing to ensure the metal in contact with the fluid possesses a smooth surface. Moreover, the calculations according to BS EN 13480‑3:2017 suggest the design configuration is able to withstand a theoretical maximum working pressure of 9.08 MPa^20^.

### Simulation of electric field within the resistivity cell

The electric field within the resistivity cell was calculated using a simplified model on the COMSOL Multiphysics (Version 5.4, Stockholm, Sweden) software with the finite element method. The simulation was used to determine the length of the guard electrodes when facing the cathode, at which the measuring volume would possess a homogenous electric field. The minimum length was determined to minimise the volume of fluid required for each test and was found to be around 5 mm at each end of the measuring electrode. The boundary conditions were assigned to the model, with the anode assigned a 500 V electric potential and the other cell components outside the fluid being grounded. The maximum electric field strength determined by the simulation within the cell was around 600 kV/m, much higher than the average field strength of 250 kV/m within the measuring volume. The model was meshed and solved for its electric potential distribution in the geometry. Poisson's equation for electrical potential (φ), denoted in Eq. (7), was solved with the potential difference across the electrodes as the boundary condition^21^.

7 $$\nabla^{2} \varphi = - \frac{{\rho_{f} }}{\varepsilon }$$

${\rho }_{f}$ is the free charge density, assumed to be zero, and ε is the permittivity of the test fluid. The solution gave the potential distribution, which on differentiation provided the electric field distribution $E$ according to Eq. (8):

8 $$\nabla E = \frac{\rho }{\varepsilon }$$

The simulation was performed assuming that space charges were absent and the resistivity of the liquid was homogenous. The electric field is homogenous within the measuring volume while being significantly distorted at the bottom region between the electrodes and the EPDM seal and at the top of the anode, as shown in Fig. 1C and D respectively. The high electric field strengths resulting from the bending of the electrode can lead to an early electrical breakdown during resistivity measurement and hence must be considered when performing the tests. This maximum field strength during a test may be higher in real life due to the possible formation of charge layers around the measuring electrode, which distort the field and affect the current flowing through the measuring electrode^22^.

### Experimental setup

#### Preparation of containers

The 500 ml whitey cylinders were pre-dried in a drying oven set at 102 °C and left overnight to remove any contaminants, including moisture. They were then removed from the oven, set to one side, and allowed to cool. They were then fitted with a blank at one end and a ball valve at the other (so that long syringe needles could be inserted directly into the centre of the cylinder). They were then evacuated, filled with either 152a or 134a to ‘condition’ the metal surface with the existing moisture contained within the propellants, and left overnight in the lab at room temperature.

#### Preparation of dried ethanol

A molecular sieve (50 g, 3 angstroms) was placed in a suitable container, connected to a 400 ml/min nitrogen purge, and put into an adapted drying oven. The oven was switched on, and the temperature was ramped up at 60 °C per hour to 300 °C to dry and activate the sieve. The oven was then switched off and allowed to cool. A bottle of absolute fresh ethanol was opened, and water content was pre-determined via colorimetric titration (Karl Fischer titration) by injecting it into a Mitsubishi Chemical Analytech CA-310 moisture metre in accordance with ASTM-D1533^23^. A small amount of the activated sieve was added to an amber glass bottle and then filled with absolute ethanol. The bottle was then shaken by hand before being stored in a desiccator overnight in the lab. The moisture content of the ethanol was re-determined by injecting it again into the Karl Fisher apparatus. If the ethanol was not sufficiently dry, then more of the pre-dried molecular sieve was placed into another amber glass bottle, and the dried ethanol was decanted into the bottle and left in a desiccator overnight.

The moisture content was then analysed using the Karl Fisher apparatus, and the clear liquid was decanted into a glass sample bottle fitted with a crimp-type septum. It was stored in a desiccator until needed.

### Preparation of samples

#### Ethanol samples

The sample cylinder was evacuated, and a septum was added to the ball valve beneath a Swagelok nut. The required amount of ethanol was measured in an appropriately sized microlitre syringe, and the weight was recorded before injecting it into the cylinder through the septum and valve into the cylinder. The syringe was re-weighed, and the weight difference injected was recorded. The septum was detached, and the cylinder was plunged into liquid nitrogen for one minute. It was then weighed before attaching a 134a and 152a supply cylinder. The valves were opened, and liquified propellant was transferred into the cylinder. The sample cylinder was detached and re-weighed to determine the amount of 134a transferred. If the weight recorded was below the required amount, the transfer method was repeated until the correct amount of 134a had been deposited into the sample cylinder. The cylinder was put aside for four days to reach room temperature and to condition the metal surface again before analysing the contents for moisture.

#### Moisture samples

This preparation was similar to the procedure described above, with the exception that water was delivered into the cylinder via a microliter syringe with the liquefied propellant. If the moisture content was below the levels calculated, the cylinder contents would be emptied, and the procedure would be repeated. The water concentration in this study is expressed as parts per million (ppm), defined with respect to the mass.

### The methodology of performing the resistivity tests

To calculate the resistivity, a voltage of 100 V DC was applied to the 2-mm gap for 10- and 60-min intervals, leading to an average field strength of about 50 kV/m, with the final current being used after a steady state had been achieved, indicating that the charge carriers have reached dynamic equilibrium.

A voltage of 100 V was applied due to void the dielectric breakdown, safety concerns, and to comply with the IEC standards. Initially, the current was measured for 10-min intervals before increasing the interval to an hour. The HFCs were also investigated at commercial-grade purity, with > 99.9% for R134a, R227ea, and R152a. However, it should be noted that these propellants are hygroscopic, and both commercial and pharmaceutical-grade samples may contain about 10 ppm of moisture. The procedure used involved purging the cell with nitrogen before vacuum pumping the cell with an ultimate vacuum pressure of 6 Pa (4.5 × 10^–2^ Torr) and then inserting the liquid propellant. The measured values allow one to easily discern whether the propellant is in its gaseous or liquid state, as they differ by orders of magnitude.

**Figure 2:**
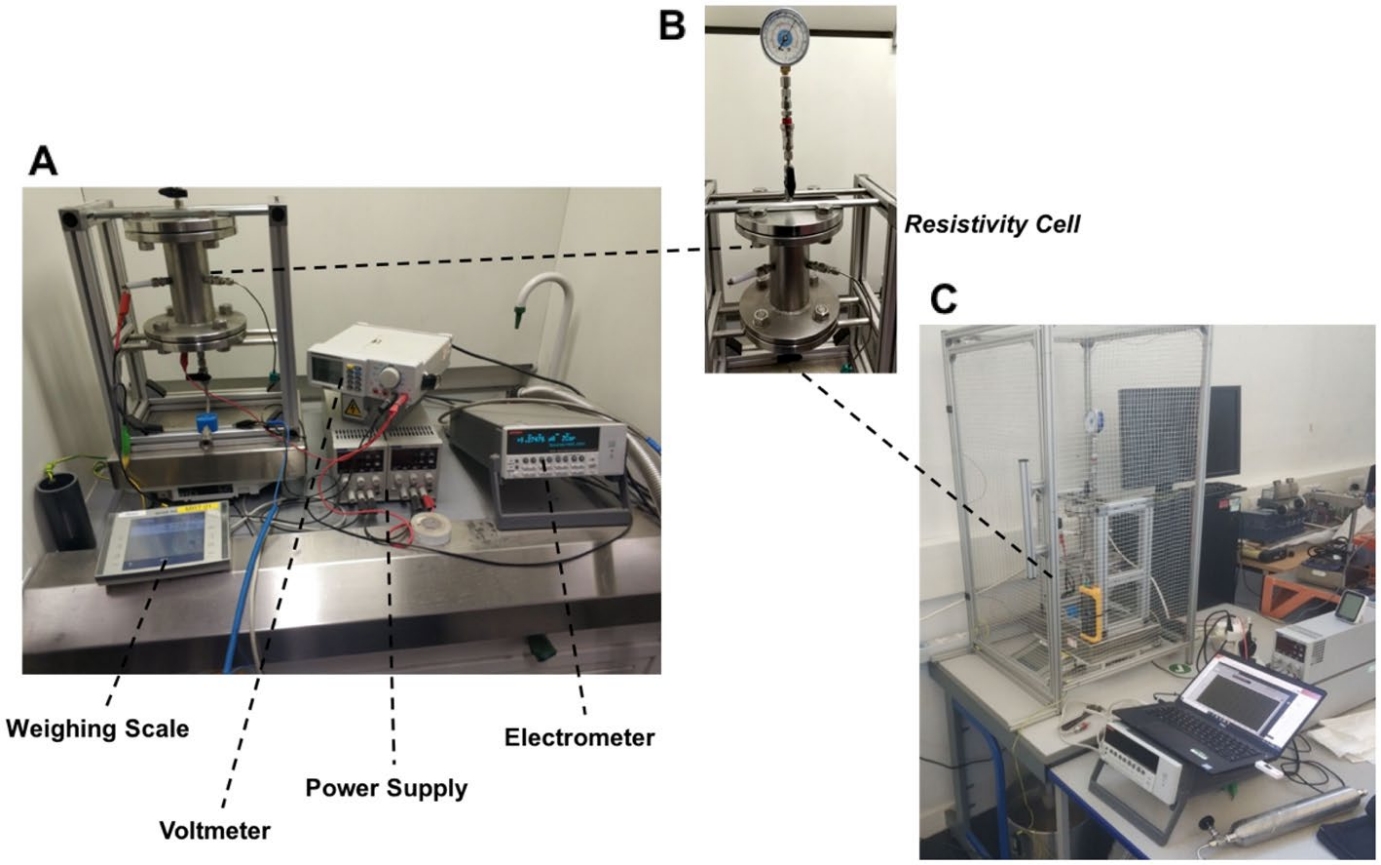
(**A**) The experimental setup of the resistivity measurement system and associated equipment, (**B**) Resistivity cell and manometer, and (**C**) Protective cage enclosing the resistivity cell.

**Figure 3:**
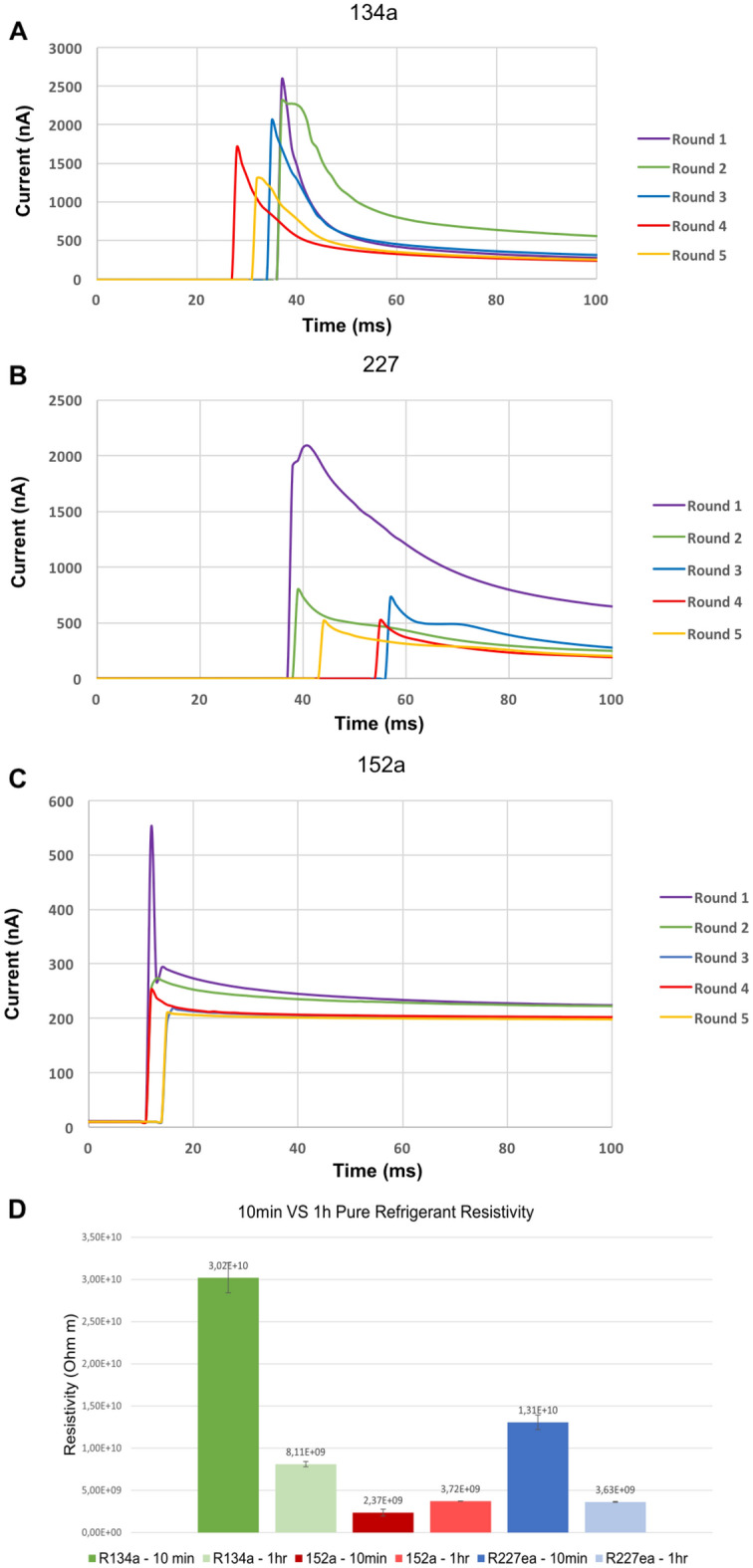
Graphs showing the change in current over time for each of the propellants for each test that is completed, including (**A**) R134a, (**B**) R227ea, and (**C**) R152a. The average room temperature for these measurements was about 20 °C. **(D)** Bar graph showing the mean resistivity values for each of the pure propellants measured at 10-min and 1-h intervals.

A manometer in the form of a refrigerant gauge was connected to the top flange, as shown in Fig. 2A–C. It was used to monitor the cell's pressure and temperature to ensure the propellant was in the liquid phase. The pressure was used to determine the temperature of the liquid using a temperature–pressure correlation embedded within the pressure gauge. 100 V DC with a positive polarity was applied via an external voltage source for a set interval. The current was recorded every 0.1 seconds using a Keithley Electrometer model 6517B with a 10 atto amps (aA) current measurement resolution and a ± 0.2% accuracy at the 2 nA range. The electrometer was connected to a laptop with a custom LabVIEW (Version 2023 Q1, Austin, US) program (Supplementary Fig. S1: Custom LabVIEW virtual instrument (VI) developed to program electrometer and record measurements) to run and record the results. The virtual instrument was designed to automate the data acquisition and allows one to observe the current against time in a graph format in real time to observe the performance. It allows one to adjust the settings, including the measurement delay and the number of measurements. An image of the experimental setup is shown below (Fig. 3). This was then repeated five times for each propellant at each time interval and three times for each moisture and ethanol concentration. The methodology for performing the tests is outlined as follows:

A transfer cylinder (500 ml) was filled to its maximum safe volume (400 ml) with propellant. The transfer cylinder was evacuated with a vacuum pump before filling. Then the cylinder was briefly chilled in liquid nitrogen to cool, using thermal gloves and goggles. The transfer cylinder was then filled from a larger cylinder before weighing it. The cell was then purged with nitrogen gas for 10 min, which helped to remove moisture and oxygen. The cell was then evacuated with a vacuum pump for 30 min using 10^–2^ Torr pressure. The transfer cylinder was then placed in a water bath at 29 °C. It was left to acclimatise for between 30 and 60 min. An earth strap was attached to the cylinder and then filled with at least 50 ml of propellant from the transfer cylinder via the bottom fitting until the pressure exceeded the saturation vapour pressure.

A scale was used to determine the volume being filled. The pressure and temperature were measured from the refrigerant gauge, and the cell wall temperature was noted down using a RS pro 206-3722 temperature sensor. The electrometer and voltage source was connected to the cell using crocodile clips, and 100 V was applied from the voltage source. The electrometer was connected to the LabVIEW (Version 2023 Q1, Austin, US) program on the laptop, and the current was measured over time. The final current measurement value was used to calculate the resistivity; after the initial peak, the current decayed and reached a steady state where the charge carriers reached dynamic equilibrium. The tests were measured and repeated at least three times at room temperature (21–23 °C) and various humidities in the lab. The propellant was then evacuated into a ventilated fume hood. The device was enclosed in a grounded metallic cage that served as a Faraday cage to prevent electromagnetic fields from affecting the results. It also helped to protect the user from experiencing an electric shock during the experiment in the event of accidentally touching the electrified pipe at the bottom that connects to the anode. The cage was set up so that the power source turns off if it is opened while the voltage is on. The propellant mixtures with ethanol and water were also tested in the same way, with the mixtures tested in order of smallest concentration of ethanol (134a: 0, 0.1, 0.3, 0.96, 3.8, 7.2, 12.5%; 152a: 0, 0.1, 0.3, 1, 3.9, 6.1%) or water (134a: 46, 174, 250, 500 ppm; 152a: 50, 277, 448, 896 ppm) to highest. Before testing each concentration, the cell was purged with nitrogen, and the vacuum pump was used to empty the cell.

## Results and discussion

### The resistivity of pure propellants

The electrical resistivity was determined for three of the most widely used substitutes for CFC and HCFC propellants. After the DC voltage was applied, the recorded current decreased after an initial spike due to polarization effects. Transient charge-carrier drift processes occur due to ions being attracted to or repelled from electrodes based on their polarity. Figure 3A–C show the recorded current plotted as a function of time in the case of each propellant for a set temperature (~ 20 °C). The first peak occurs after a few ms when the charge carriers in the dielectric liquid migrate from one electrode to the other. This duration is known as the time of flight (ToF).

Our tests have shown that stable measurement values could be taken after 10 min and that extended stress does not lead to significant further change. The tests are labelled by the order in which they were performed. It can be seen that the current values measured tend to decrease slightly as the order of the tests increases. This may be because the cell gets flushed with each test with the propellant, and some impurities get removed, leading to measured smaller current values. The measurements were repeated at least five times. Similar diagrams are observed for each of the propellants. The graphs tended to peak with a few hundred nA, with R134a tests peaking at higher currents when the voltage source was switched on at around 2000 nA before stabilising at approximately 50 nA. The 134a tests peaking at higher current values may be because these tests were conducted earlier than the other propellants; therefore, contaminant concentrations may have been slightly higher before being flushed after repeated trials. Initial tests for each propellant tended to peak much higher and settle at slightly higher current values after 10 min. It may be wise to repeat the tests more times until the readings stabilise and discard initial test results due to contaminant fears to attain more reliable results.

The ToF, is determined from the time interval between when the voltage is applied first to the moment of the peak current. The value obtained for all three pure propellants is 1 ms, limited by the time resolution of the current measurement. The charge carrier mobility is then calculated as $\mu = \frac{{L^{2} }}{ToF \cdot V} = \frac{{2\;{\text{mm}}^{2} }}{{1\;{\text{ms}} \cdot 100\;{\text{V}}}} = 4 \cdot 10^{ - 5} \frac{{{\text{m}}^{{2}} }}{{{\text{Vs}}}}$. And the drift velocity of the pure propellants is $v = \mu \cdot E = 2\frac{{\text{m}}}{{\text{s}}}$, with a significant uncertainty of at least 50%.

### Resistivity for different measuring intervals

Figure 3A–D shows the differences between the resistivity values for different measuring times and various types of propellants. It can be seen that the resistivity for the 10-min values tends to be significantly greater than the one-hour values; this is due to the number of charged particles increasing thereby leading to a downward drift in resistivity. The likeliest mechanism for this is the fate of the charge carriers produced early in the reading is not to disappear once discharged, but to leave some residual fragments that can add to the pool of potential charge carriers, so that the pool of charge carriers keeps increasing with time in the run. This was, however, not the case for the 152a propellant, which demonstrated the opposite relationship, with the 1-h average value for resistivity being slightly larger. Here additional charge carriers are not being created after the original one’s discharge at the electrodes. Instead, the pool of carriers remains roughly constant, with a slight reduction through the run leading to slightly rising resistivity. This could be accounted for by either the carriers surviving discharge to 'go around and do it again' as a semi-stable population (i.e. an initial positively charged carrier goes to the cathode, and pick up two electrons thus becoming a negatively charged carrier, goes to the anode, loses two and so on) or being discharged and lost from the carrier population to be replenished by an ionisation process (e.g. the ionisation of water) maintaining a pseudo-constant population.

**Figure 4:**
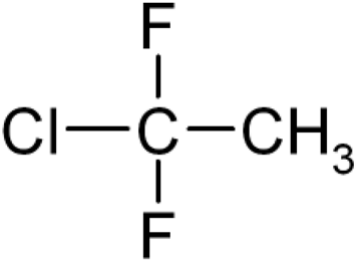
Spatial configuration of R152a, also known as 1,1-Difluoroethane.

The 227ea and 134a were found to have a resistivity at least one order of magnitude higher than 152a. This is expected to result from the chemical structure of 152a being C_2_H_4_F_2_ (shown in Fig. 4) where there is a strong attraction between water molecules and the two fluorine atoms due to the high electronegativity of fluorine. When C_2_H_4_F_2_ interacts with water, the highly electronegative fluorine atoms attract the partial positive charges on the hydrogen atoms of water molecules, leading to strong dipole–dipole interactions. This makes the 152a much more susceptible to water contamination.

The tests were repeated at least three times due to the limited number of samples available. The resulting values of the resistivity for pure R134a, R227ea, and R152a, defined as the mean of the 10-min interval measurements, are (3.02 ± 0.18) ∙ 10^10^ Ωm, (1.31 ± 0.08) ∙ 10^10^ Ωm, and (2.37 ± 0.4) ∙ 10^9^ Ωm, respectively. The uncertainties quoted are the standard deviations from the set of measurements taken.

Several small instrumental uncertainties limit the measurement. There is a 0.2% uncertainty in the current measured by the electrometer; the cell constant has an assumed uncertainty of 0.5%; and the applied voltage value has an uncertainty of 1%. Using Gaussian error propagation, the resistivity has a total instrumental uncertainty of about 1.1%. This is smaller than the standard deviations quoted above; the measurement is, therefore not dominated by instrumental uncertainties.

### Comparison of measured resistivity to other published results

Table 2 compares the results to data from other published literature. Feja^9^ analysed liquid R134a at varying temperatures. His results indicate that the resistivity of R134a is temperature-independent and in the order of 10^8^ Ωm. However, our measured values are on the order of 10^10^ Ωm for ambient temperatures and saturation pressures for liquid phase measurements. A similar trend is observed when comparing the results to those obtained by Fellows et al.^8^. The measurements for R152a, which were performed to serve as a reference, correspond well to existing data. However, the measured value of the DC resistivity for R134a is about a factor of 45 higher.

**Table 2:** The measured resistivity values [Ohm m] obtained from the 10-min intervals, compared to values quoted in other literature.

Propellant	This measurement	Fellows et al.^8^	Feja^9^	Dschung^10^
R134a	3.02 × 10^10^	6.6 × 10^8^	~ 10^8^	6.6 × 10^6^
R152a	2.37 × 10^9^	2.2 × 10^9^	~ 10^7^	n/a
R227ea	1.31 × 10^10^	n/a	n/a	1.3 × 10^8^
Electric filled values				
Electric field strength (kV/m)	50	250	250	250
Time interval (min)	10	Not provided	1	60

The differences are discussed in the text.

As the resistivity is highly dependent on the water content and other impurities of the tested substance and the value of the applied field strength, such deviations are not unusual. The maximum water content present in our propellants, according to the manufacturer’s specifications, is about 10 ppm. As described above, the measurement’s uncertainties are assumed to contribute further to the detected deviations.

Maintaining the quality of the test fluid sample is very important because its electrical properties are susceptible to contamination and impurities. This is one of the reasons why it is very hard to compare measurements from various researchers because of differences in the purity level of the test fluid sample. Also, comparing properties between researchers can be difficult because the temperature and pressure are not always given.

There are several reasons for these significant discrepancies; one is possible impurities. Water content, for example, strongly influences the resistivity; the resistivity decreases when water is added. This study prepared the samples very carefully to minimise moisture contamination. Also, before the propellants were inserted into the cell, the transfer was handled with additional precautions: a heat gun was used on the transfer cylinder, and its nuts were loosened to evaporate and flush out any residual moisture. Moreover, no plastic pipes were used as all these HFAs are considered highly hygroscopic and can even make plastic parts more likely to introduce water via diffusion phenomena. The maximum water content present in our propellants, according to the manufacturer’s specifications, is at most 10 ppm.

The resistivity also tends to be a strong function of the electric field. Lower test voltages can be another reason for higher resistivity measurements compared to previous researchers. But in this measurement, applying voltages above 100 V was impossible due to spacing between anode and cathode (2-mm) to avoid dielectric breakdown.

Additionally, there are differences in the experimental setup. Feja^9^ used a modified cell arrangement according to IEC 60247 without guard ring. The absence of a guard ring forced him to calibrate the test cell with a well-known liquid in advance.

To summarise, several factors make comparisons to other experiments difficult: the purity level of the test fluid sample directly influences electrical properties, but temperature, pressure, and applied voltage values also have an impact. The improved and custom-designed cell used for this measurement is also likely to contribute to a more accurate result than the literature.

### The resistivity of mixtures of propellants with water or ethanol

Fellows et al.^8^ and Feja^9^ studied the DC resistivity of pure R134a and R152a. However, this study presents further information regarding the resistivity of mixtures of propellants with moisture and ethanol.

**Figure 5:**
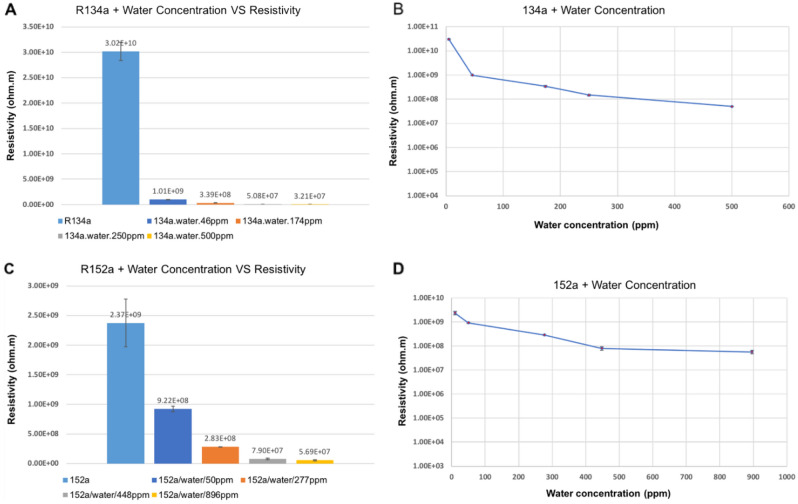
Graphs showing the resistivity values of 134a and 152a propellant mixtures with different concentrations of water: (**A**) 134a + water, (**B**) line graph of 134a + water, (**C**) 152a + water, and (**D**) line graph of 152a + water. The average room temperature for the set of measurements with R134a was 23.6 °C, and the average room humidity was 39.7%. For the measurements taken with 152a, the average room temperature was 27.6 °C, and the average room humidity was 42%.

Figure 5 shows the resistivity values for the 152a and 134a propellants with different water concentrations (134a: 46, 174, 250, 500 ppm; 152a: 50, 277, 448, 896 ppm). Moisture content will ionise in the electrical field, meaning the water molecules undergo electrolysis and split into hydrogen ions H^+^ and hydroxide OH^−^. The hydrogen ions react with the water molecules at a low rate, forming hydronium ions, H_3_O^+^. These charge carriers reduce the resistivity and will then respond with the electrons from the cathode and anode, forming dihydrogen H_2_ and water H_2_O and oxygen O_2_.

The values for 134a water concentrations can be seen to decrease exponentially in resistivity with increasing water concentrations before levelling off at 500 ppm of moisture. There is an initial significant decline in resistivity between the pure propellant and the addition of 46 ppm of moisture, resulting in order of magnitude decrease in resistivity. The graph for 152a and moisture shows a similar pattern with similar resistivity values for the different moisture concentrations, where the concentration of 900 ppm of moisture showed a resistivity value that had plateaued relative to the 500 ppm value. This plateau shows that added water concentrations may no longer significantly affect the resistivity of the mixture. This may be due to added water concentrations that cause the resistivity to approach the value for water.

As has already been observed in Fig. 5, 134a has about an order of magnitude higher resistivity than 152a, even at similar water concentration levels. This is understood to be related to the different ionic mobility. The dipole moments of the propellants are 2.06 Cm for 134a and 2.26 Cm for 152a, which means that 152a is a more powerful dipole.

The HFC molecules cluster around the charged ions, but for 152a the ionic solvated cluster is smaller than that for 134a. This means that drag against the rest of the liquid medium as the ion moves in the cell field will be less, and the ionic velocity will be higher, leading to a higher cell current and lower resistivity for 152a.

**Figure 6:**
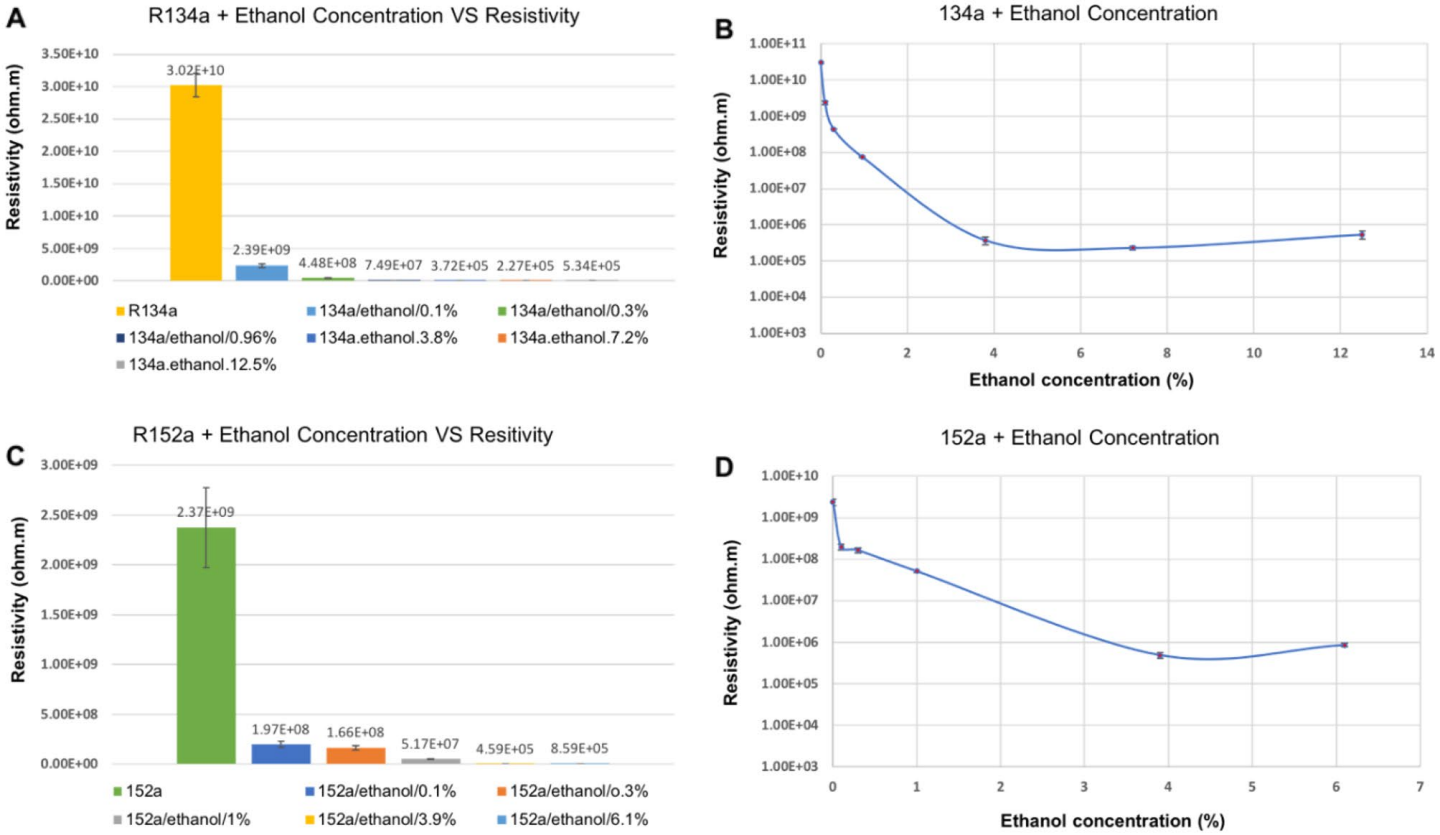
Graphs showing the resistivity values of 134a and 152a propellant mixtures with different concentrations of ethanol: (**A**) 134a + ethanol, (**B**) line graph of 134a + ethanol, (**C**) 152a + ethanol, and (**D**) line graph of 152a + ethanol. The average room temperature for the set of measurements with R134a was 24.5 °C, and the average room humidity was 40.3%. For the measurements taken with 152a, the average room temperature was 25.4 °C and the average room humidity was 47%.

Figure 6 shows the resistivity values for the 152a and 134a propellants with different ethanol concentrations. Trace moisture can influence these studies, but due to the precautions taken, the moisture concentration is less than 10 ppm and, therefore, will have a negligible impact compared to the dominant effect of ethanol. It is expected that ethanol will be ionised in the electrical field via the reaction^24^:

$${\text{CH}}_{{3}} {\text{CH}}_{{2}} {\text{OH}} \to {\text{CH}}_{{3}} {\text{CH}}_{{2}} {\text{O}}^{ - } + {\text{ H}}^{ + }$$

and the hydrogen ion then instantly associates with non-ionised ethanol via:

$${\text{H}}^{ + } + {\text{ CH}}_{{3}} {\text{CH}}_{{2}} {\text{OH}} \to {\text{CH}}_{{3}} {\text{CH}}_{{2}} {\text{OH}}_{{2}}^{ + }$$

The two ions CH_3_CH_2_OH_2_^+^ and CH_3_CH_2_O^−^ will lower the energy of the carried charge by virtue of the larger molecule, providing a considerable redistribution of the charge by polarising the bonds within the molecule. Ethanol charge dissipation is more potent than HFC solvation, meaning at low ethanol concentrations (less than 1%), the ions are surrounded by a much smaller HFC solvent cage, which is also more tenuously attached. But when ethanol concentration rises, a ‘hybrid’ solvation is formed, where both HFC and neutral ethanol molecules make up the solvation cage.

As displayed in Fig. 6, the resistivity drops with an increasingly large ethanol concentration. However, the resistivity plateaus at a concentration of approximately 4% and then slowly rises again. At ethanol levels below 4%, there is an increase in charge carriers, a slight decrease in cage sizes, and less drag on the HFA carrier medium, leading to an increase in current and a reduction in resistivity.

An explanation for the plateau and subsequent rise of resistivity for larger ethanol concentrations can be found by assuming that the rate at which negative ethanol ions are created slows down and is no longer proportional to the ethanol concentration.

Instead, hydrogen bonding will occur with an increased presence of ethanol molecules, both for neutral ethanol molecules and for ethanol ions. Hydrogen bonding^25^ is a weak form of coupling between polar molecules; in this case, the partially negatively charged oxygen ion will bond with the partially positively charged hydrogen ion of another ethanol molecule. Hydrogen bonding is enhanced for the negatively charged ethanol ion CH_3_CH_2_O^−^ because the oxygen carries an even larger partial negative charge.

Consequently, larger clusters of charge carriers and a larger solvent cage are formed, and their ionic mobility and drift velocity drop; hence, this new dynamic state increases the resistivity of the propellant mixture. Further studies with varying temperatures and larger sample sizes should be conducted to more precisely estimate the concentration at which the plateau and turn-around effects occur.

The ToF for the mixtures was estimated as previously for the pure propellants by determining the time interval between when the voltage was switched on and when the peak current occurred. The current as a function of time was measured at least three times for each concentration value. However, the result is again limited by the time resolution of the measurement and was therefore again determined as 1 ms, leading to the same drift velocities of $v = 2\frac{{\text{m}}}{{\text{s}}}$ with a considerable uncertainty of at least 50%.

**Figure 7:**
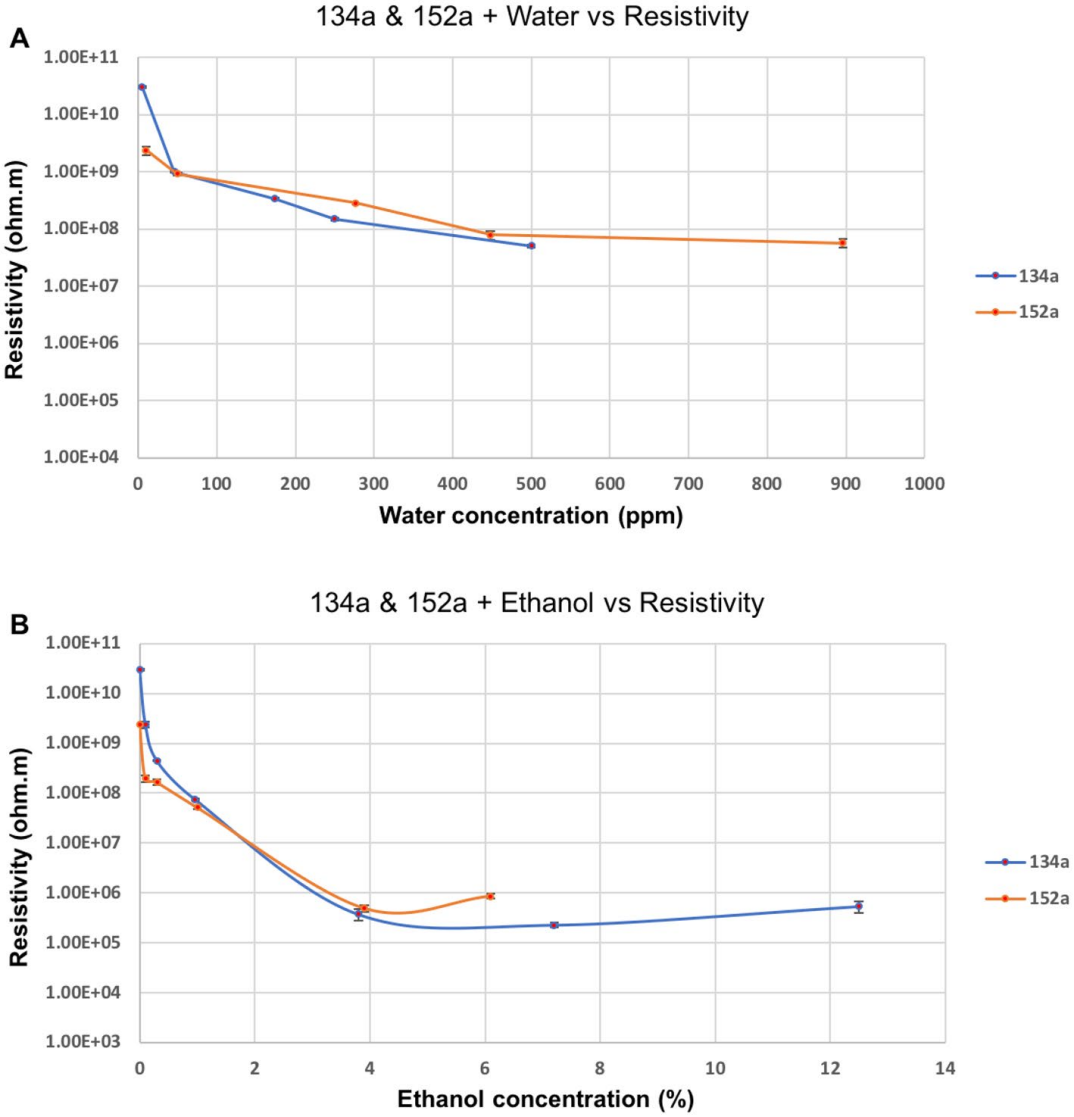
Graphs showing the change in resistivity for two different propellant mixtures with (**A**) different concentrations of water and (**B**) ethanol.

In Fig. 7, the measured resistivity graphs as a function of either the water or the ethanol concentration are superimposed for propellants 134a and 152a, indicating a similar behaviour for both materials.

Knowing precisely how moisture and ethanol content influence resistivity can help improve medical inhaler formulations and increase their effectiveness. The resistivity of the propellant used in MDIs must be controlled and maintained at a fixed value; otherwise, an electrical charge will accumulate, which will prevent the uniform dissipation of the drug aerosol and limit the deposition of the pharmaceutical in the human lungs. More studies are needed to quantify the dependence of the electrical properties of propellants on a variety of conditions, for example, different temperatures and air humidity levels.

Furthermore, the studies performed with 152a are crucial to assessing its potential to be used as a propellant in MDIs, replacing 134a and 227ea in the future. The risk of being ignited during charge accumulation must be minimised, and therefore the resistivity properties of the pure substance and in mixture with water and ethanol have been extensively investigated. The sample provided by the sponsoring pharmaceutical company and we have no control over the range for extension. That is the reason the values for the range are different.

## Conclusion

In conclusion, the resistivity of propellants, including 134a, 152a, and 227ea, and their mixtures with different concentrations of ethanol and water were examined using a novel device developed for this study. The measured resistivity values for pure R134a, R227ea, and R152a were found to be 3.02 × 10^10^ Ωm, 1.31 × 10^10^ Ωm, and 2.37 × 10^9^ Ωm, respectively. The resistivity values are generally higher than those quoted in the literature, which is attributed to several factors, such as higher purity, custom cell design, and an improved experimental process. The resistivity was also measured for mixtures of propellants 134a and 152a with different water and ethanol concentrations. It was observed that the resistivity dropped with increased moisture content. The dependence of the resistivity on the ethanol concentration was found particularly interesting. The measurements showed that adding ethanol caused a significant reduction in resistivity values for mixtures up to around 4% concentration, after which the resistivity values plateaued and even began to increase. This suggests that a specific concentration range of ethanol can significantly alter the electrical properties of these propellants. The reason for that is the formation of large molecule clusters and solvent cages that limit the mobility of charge carriers and thus increase the electrical resistivity.

The findings of this research can contribute to developing more efficient and effective propellants for various industrial applications, such as metered-dose inhalers. Precise knowledge and control of the resistivity can avoid charge effects, which would be detrimental to consistent lung deposition of drug aerosols. Future studies may investigate the effects of other additives and their concentration ranges on the resistivity of propellants to enhance their properties for specific applications. These measurements are experimentally very challenging. It is crucial to minimise the contamination of the samples with moisture, and the pressure in the cell must remain stable to ensure the propellants stay in liquid form, avoiding vapour formation. Therefore, improvements to the experimental setup should also be the subject of future studies.

### Supplementary Information

Supplementary Figure S1.

## Data Availability

The datasets used and/or analysed during the current study are available from the corresponding authors on reasonable request.
