# Supplementary material for: Liquid resistivity of pharmaceutical propellants using novel resistivity cell
Source: Sci Rep. 2023 Nov 5;13:19122. doi: 10.1038/s41598-023-45253-6 (PMC10625955; doi:10.1038/s41598-023-45253-6)
Supplement: Supplementary file 1 — Supplementary Figure S1. [file 41598_2023_45253_MOESM1_ESM.pdf]

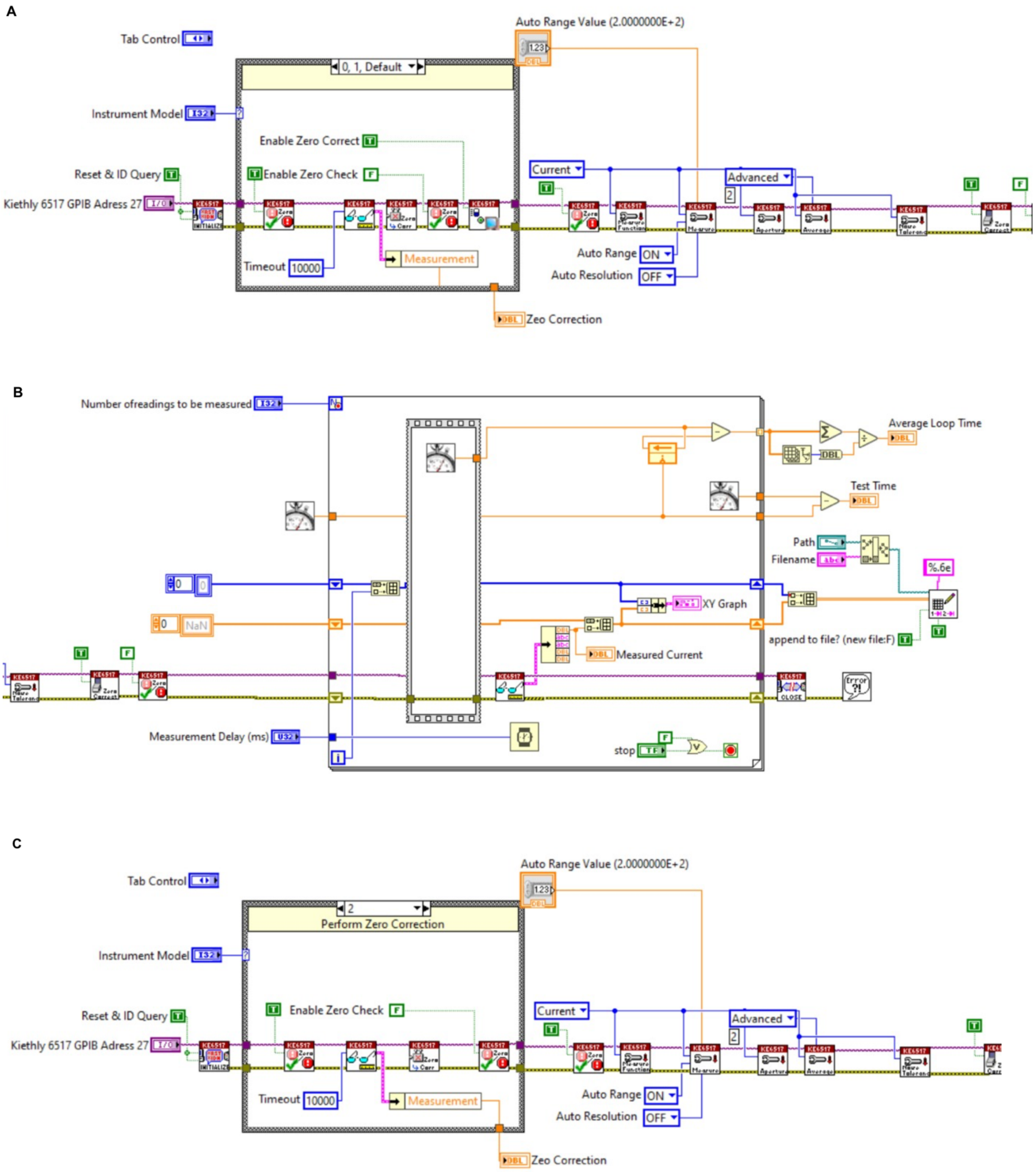

Figure S1. Custom LabVIEW (Version 2023 Q1, Austin, US) virtual instrument (VI) developed to program electrometer and record measurements. The current is measured as a function of time and displayed in a graph, the measurement delay and time period parameters can be adjusted. (**A**) one part of the block diagram of the VI that was developed. (**B**) another section of the block diagram and (**C**) block diagram for an alternative setting for the zero-correction function.
